# Challenges of sperm cryopreservation in transferring heat adaptation of corals across ocean basins

**DOI:** 10.7717/peerj.13395

**Published:** 2022-05-27

**Authors:** Emily J. Howells, Mary Hagedorn, Madeleine J.H. Van Oppen, John A. Burt

**Affiliations:** 1 Water Research Center and Center for Genomics and Systems Biology, New York University Abu Dhabi, Abu Dhabi, United Arab Emirates; 2 National Marine Science Centre, Faculty of Science and Engineering, Southern Cross University, Coffs Harbour, New South Wales, Australia; 3 Center for Species Survival, Smithsonian Conservation Biology Institute, Smithsonian Institution, Free Royal, Virginia, United States of America; 4 Hawaii Institute of Marine Biology, University of Hawaii, Kaneohe, Hawaii, United States of America; 5 School of Biocsiences, The University of Melbourne, Melbourne, Victoria, Australia; 6 Australian Institute of Marine Science, Townsville, Queenslabd, Australia

**Keywords:** Coral, Cryopreservation, Selective breeding

## Abstract

Reef-building corals live very close to their upper thermal limits and their persistence is imperiled by a rapidly warming climate. Human interventions may be used to increase the thermal limits of sensitive corals by cross-breeding with heat-adapted populations. However, the scope of breeding interventions is constrained by regional variation in the annual reproductive cycle of corals. Here we use cryopreservation technology to overcome this barrier and cross-breed conspecific coral populations across ocean basins for the first time. During regional spawning events, sperm samples were cryopreserved from populations of the widespread Indo-Pacific coral, *Platygyra daedalea*, from the southern Persian Gulf (maximum daily sea surface temperature of 36 °C), the Oman Sea (33 °C), and the central Great Barrier Reef (30 °C). These sperm samples were thawed during a later spawning event to test their ability to fertilize freshly spawned eggs of *P. daedalea* colonies from the central Great Barrier Reef. Average fertilization success for the Persian Gulf (9%) and Oman Sea (6%) sperm were 1.4–2.5 times lower than those for the native cryopreserved sperm from Great Barrier Reef (13–15%), potentially due to lower sperm quality of the Middle Eastern sperm and/or reproductive incompatibility between these distant populations. Overall, fertilization success with cryopreserved sperm was low compared with fresh sperm (>80%), likely due to the low motility of thawed sperm (≤5%, reduced from 50% to >90% in fresh sperm). To evaluate whether cross-bred offspring had enhanced thermal tolerance, the survival of larvae sired by Persian Gulf cryopreserved sperm, Great Barrier Reef cryopreserved sperm, and Great Barrier Reef fresh sperm was monitored for six days at ambient (27 °C) and elevated (33 °C) temperature. Against expectations of thermal tolerance enhancement, survival of larvae sired by Persian Gulf cryopreserved sperm was 2.6 times lower than larvae sired by Great Barrier Reef fresh sperm at 33 °C (27% versus 71%), but did not differ at 27 °C (77% versus 84%). This lack of enhanced thermal tolerance was unlikely due to outbreeding depression as survival was equally poor in larvae sired by Great Barrier Reef cryopreserved sperm. Rather, follow-up tests showed that cryoprotectant exposure during fertilization (0.1% DMSO) has a negative effect on the survival of *P. daedalea* larvae which is exacerbated at elevated temperature. Collectively, our findings highlight challenges of breeding corals for enhanced thermal tolerance using cryopreserved sperm, which may be overcome by methodological advances in the collection and preservation of high-quality motile sperm and minimizing the exposure time of eggs to cryoprotectants.

## Introduction

Rising sea temperatures caused by climate change are increasingly exceeding the upper thermal limits and fitness optima for reef-building corals (Hughes et al., 2018a; Jurriaans & Hoogenboom, 2019). This is evidenced by temperature driven bleaching and mortality events (Hoegh-Guldberg, 1999; Hughes et al., 2018b), increases in disease prevalence (Bruno et al., 2007; Howells et al., 2020; Randall & Van Woesik, 2015), declines in coral growth (Anderson et al., 2017; Cantin et al., 2010; Mendes & Woodley, 2002), and reductions in reproductive output (Baird & Marshall, 2002; Howells et al., 2016b; Mendes & Woodley, 2002; Ward, Harrison & Hoegh-Guldberg, 2002). While coral populations have likely undergone some adaptation to contemporary warming (Berkelmans, 2009; Fox et al., 2021; Guest et al., 2012; McClanahan & Muthiga, 2014; Pratchett et al., 2013; Sully et al., 2019), the rapid rate and extent of sea temperature rise over the coming decades threatens to exceed their adaptive capacity (Hoegh-Guldberg et al., 2018). The potential for ongoing adaptation to climate warming is greatest when heat tolerant alleles are already present within populations or can easily spread among populations by migration, and when rates of warming are limited by emissions reductions (Bay et al., 2017; Matz et al., 2018; Matz, Treml & Haller, 2020). However, because sea temperatures will continue to rise until at least 2050 under all emissions scenarios (IPCC , 2021), interventions may be required to conserve and restore populations, species, and the ecosystem services they provide (Anthony et al., 2017).

Selective breeding of corals can be applied to accelerate adaptation by increasing the frequency of heat-tolerance alleles in target populations and as such enhance thermal tolerance traits (Van Oppen et al., 2015; Voolstra et al., 2021). This involves cross-breeding individuals from a target population with heat tolerant genotype(s) sourced from the same (Drury et al., 2021; Humanes et al., 2021) or a different population, typically from a warmer location (Dixon et al., 2015; Howells et al., 2021; Quigley et al., 2020). When information on individual genotypes and phenotypes is unknown, cross-breeding with corals from a warmer location can enhance heat tolerance. For example, we recently showed that crossing Oman Sea corals with conspecifics in the warmer Persian Gulf produced offspring with thermal tolerance up to 37% higher than purebred Oman Sea offspring (Howells et al., 2021). Coral populations from warmer locations tend to carry a larger proportion of heat tolerance alleles due to local adaptation (Bay et al., 2017; Jin et al., 2016) and this host genetic background strongly contributes to phenotypic variation in coral thermal tolerance (Dziedzic et al., 2019; Fuller et al., 2020; Kirk et al., 2018; Palumbi et al., 2014). Additional benefits of population inter-breeding include increasing overall genetic diversity (Quigley, Bay & Van Oppen, 2019) and minimizing genotype-environment and phenotype-environment mismatches that occur when heat-tolerant adult corals are transplanted from warmer to cooler locations (Coles & Riegl, 2013; Howells et al., 2013). However, the feasibility and scalability of selective breeding is often constrained by the discrete reproductive timing of coral populations and the logistical challenges of transporting broodstock and/or gametes between locations.

Individual colonies of most coral species (>80%) release gametes into the water column on only one or a few consecutive nights per year (reviewed in Baird, Guest & Willis, 2009). This spawning activity is highly synchronized at the scale of reefs to maximize cross fertilization and is regulated by environmental cues including seawater temperature and lunar phases (Baird, Guest & Willis, 2009; Keith et al., 2016; Lin et al., 2021). However, due to spatial variation in these cues, the timing of spawning in the same species varies by lunar night across nearby reefs (Babcock et al., 1986), by month across regions (Howells et al., 2014; Willis et al., 1985), and by season across ocean basins (Guest et al., 2005; Keith et al., 2016). As the viability of coral gametes declines within hours of release, crosses are restricted to colonies spawning on the same night. Even when colonies from different populations produce mature gametes during the same lunar cycle, their exact time of spawning can be difficult to predict. However, mismatches in the reproductive timing of coral populations targeted for selective breeding can potentially be overcome using the cryopreservation of coral gametes.

Cryopreservation techniques for corals have been developed with the aim of conserving the genetic diversity of endangered species (Daly et al., 2018; Hagedorn et al., 2012a; Hagedorn & Spindler, 2014) and providing tools for reef restoration (Hagedorn et al., 2017). Cryopreservation techniques are the most advanced for coral sperm and have been tested on and/or used to bank genetic material for >45 Indo-Pacific and Caribbean species (https://nationalzoo.si.edu/center-for-species-survival/coral-species-cryopreserved-global-collaborators; see also Grosso-Becerra et al., 2021; Hagedorn et al., 2006; Hagedorn et al., 2012b; Ohki et al., 2014; Viyakarn et al., 2018). Cryopreserved sperm has been successfully used to breed corals from the same population across spawning nights and years (Grosso-Becerra et al., 2021; Hagedorn et al., 2017), and proof-of-concept experiments for future selective breeding between isolated populations (Hagedorn et al., 2021), but it has not yet been used for the genetic improvement of fitness traits. Here we investigate the feasibility of using cryopreserved sperm from warmer locations in the Middle East to enhance the thermal tolerance of Great Barrier Reef corals. Populations of *Platygyra daedalea* from the central Great Barrier Reef (maximum daily sea surface temperature of 30 °C) were crossed with cryopreserved sperm from distant populations in the thermally extreme Persian Gulf (36 °C) and Oman Sea (33 °C) as well as local sperm sources. We evaluated the effects of cryopreservation and sire origin on fertilization success, survival, and thermal tolerance of coral offspring.

## Materials & Methods

### Cryopreservation of sperm populations

*Platygyra daedalea* sperm was collected and cryopreserved from populations on the central Great Barrier Reef (Pelorus Island, Trunk Reef), the Oman Sea (Al Aqah Island), and the Persian Gulf (Saadiyat Reef) (Table 1). Permits for coral collection were provided by the Great Barrier Marine Park Authority (G12/35236.1), the Fujairah and Dibba municipalities, and the Environment Agency Abu Dhabi.

**Table 1:** Coral sperm samples collected from *Platygyra daedalea* populations and used for fertilization and survival experiments in this study. Population daily maximum temperatures were obtained from *in situ* loggers recording over hourly intervals between 2010 and 2014 (Howells et al., 2016a; Howells et al., 2016b) and https://www.aims.gov.au/docs/research/climate-change/climate-monitoring/sst.html. Values for Trunk Reef were obtained from neighboring Kelso Reef (16 km distance).

Usage date	Population and Region	State/Emirate and Country	Daily maximum temperature	Cryopreservation details: no colonies, date, sperm density, DMSO concentration	Fertilization: Sperm density, DMSO concentration	Pre-freeze forward sperm motility	Post-thaw forward sperm motility
21-Nov-2013 22-Nov-2013	Pelorus Island, Great Barrier Reef	Queensland, Australia	30.5 °C	5, - 4, -	3 × 10^5^ ml^−1^, 0% 1 × 10^6^ ml^−1^, 0%	–	–
21-Nov-2013 22-Nov-2013	Pelorus Island, Great Barrier Reef	Queensland, Australia	30.5 °C	5, 4-Dec-2012, 3 × 10^8^ ml^−1^, 10% 5, 4-Dec-2012, 3 × 10^8^ ml^−1^, 10%	3 × 10^5^ ml^−1^, 0.01% 1 × 10^6^ ml^−1^, 0.03%	50% to 90% in all colonies	5%
21-Nov-2013 22-Nov-2013	Trunk Reef, Great Barrier Reef	Queensland, Australia	30.5 °C	1, - 3, -	3 × 10^5^ ml^−1^, 0% 1 × 10^6^ ml^−1^, 0%	–	–
21-Nov-2013 22-Nov-2013	Trunk Reef, Great Barrier Reef	Queensland, Australia	30.5 °C	6, 4-Dec-2012, 1 × 10^8^ ml^−1^, 10% 6, 4-Dec-2012, 1 × 10^8^ ml^−1^, 10%	3 × 10^5^ ml^−1^, 0.03% 1 × 10^6^ ml^−1^, 0.09%	50% to 70% in all colonies	5%
21-Nov-2013 22-Nov-2013	Al Aqah Island, Oman Sea	Fujairah, United Arab Emirates	33.3 °C	4, 23-Apr-2013, 3 × 10^7^ ml^−1^, 10% 4, 23-Apr-2013, 3 × 10^7^ ml^−1^, 10%	3 × 10^5^ ml^−1^, 0.1% 1 × 10^6^ ml^−1^, 0.3%	50% to 90% in all colonies	5%
21-Nov-2013 22-Nov-2013	Saadiyat Reef, Persian Gulf	Abu Dhabi, United Arab Emirates	35.7 °C	7, 27-Apr-2013, 3 × 10^8^ ml^−1^, 10% 7, 27-Apr-2013, 3 × 10^8^ ml^−1^, 10%	3 × 10^5^ ml^−1^, 0.01% 1 × 10^6^ ml^−1^, 0.03%	50% to >90% in 6 of 7 colonies	5%

*P. daedalea* was identified according to the morphological criteria in Veron (2000) and following examination of the species collection at the Museum of Tropical Queensland, and only colonies with typical morphological features (*i.e.,* meandroid growth form and messy ragged septa) were targeted for collection. For each population, single fragments were collected from 4-9 colonies with visibly mature gametes (*i.e.,* pigmented eggs) prior to the predicted nights of annual broadcast spawning and were housed in aquarium facilities at the Australian Institute of Marine Science (Great Barrier Reef corals, December 2012) or New York University Abu Dhabi (Oman Sea and Persian Gulf corals, April 2013). Colony fragments were individually isolated in buckets with filtered seawater at sunset, monitored for spawning activity, and spawning dates and times were recorded in the Coral Spawning Database (Baird et al., 2021).

Spawned egg/sperm bundles were collected with transfer pipettes, and after bundles had separated, concentrated sperm was filtered from eggs with 60 µm plankton mesh. Sperm forward motility was assessed for each colony sample under a microscope immediately following collection and after 30–60 min, if initial motility was poor. Individual sperm samples with motility in ≥50% of cells were pooled by population and cryopreserved using established methods (Hagedorn et al., 2012b). Briefly, 500 µl of the pooled sperm sample from each population (in 0.2 µm filtered seawater) were added to a labelled cryotube, with gradual addition of 500 µl of 20% dimethyl sulfoxide (DMSO; final concentration 10%), followed by gentle mixing by inversion. Samples were incubated at room temperature for 10 min and then added to a custom cryopreservation rack suspended above an insulated container of liquid nitrogen. After samples were cooled to below −80 °C (∼5 min) at a mean freezing rate of 18  °C per minute, they were submerged and left floating in liquid nitrogen for a minimum of 10 min. Sperm samples were stored and transported at −196 °C using liquid nitrogen dry shippers. Samples were thawed immediately prior to use by immersion of cryotubes in filtered seawater at room temperature for ∼3 min, and were mixed gently by immersion. Post-thaw sperm motility was assessed *via* examination under a microscope in 1:10 dilutions.

### Fertilization assays

The ability of local and distant cryopreserved sperm populations to fertilize *P. daedalea* eggs was tested in a subsequent spawning season on the Great Barrier Reef. Sperm samples from the Oman Sea and Persian Gulf were imported into Australia under CITES permits 13MEW5563 and PWS2013-AU-001007, and used for breeding in a quarantine approved research facility under Department of Agriculture, Fisheries and Forestry permit IP13016887.

Fragments of *P. daedalea* colonies with visibly mature gametes were collected from the same local Great Barrier Reef populations (*i.e.,* Pelorus Island, Trunk Reef) prior to the predicted nights of spawning in November 2013, and were housed in aquarium facilities at the Australian Institute of Marine Science. Colony fragments were monitored for spawning activity and gametes were collected and separated as outlined above over two nights of spawning (21st and 22nd November). Eggs from Pelorus Island colonies (*n* = 4) and Trunk Reef colonies (*n* = 2) were washed with 0.2 µm filtered seawater to remove residual sperm (*i.e.,* six egg treatments) and divided among replicate scintillation vials (35–100 eggs in 15 ml of 0.2 µm filtered seawater, mean = 52 eggs per vial). Cryopreserved sperm from each population and fresh local sperm was added to three separate replicate vials of eggs for each maternal colony (Table 1). An additional three replicates had no sperm added to quantify any self-fertilization of each maternal colony. Sperm was provided at a final density of 3 × 10^5^ ml^−1^ on the first night of spawning, and was increased to 1 × 10^6^ ml^−1^ on the second night of spawning in an attempt to increase fertilization success. Vials were transferred to at incubator at 27 °C (ambient seawater temperature) with gentle agitation (35 rpm). After four hours, the number of fertilized eggs, observed as first-cleavage to multi-cell stages, was counted in each vial under a dissecting microscope. Additional qualitative monitoring was undertaken until 96 h, after which any living material was destroyed.

### Survival experiments

Variation in the survival of *P. daedalea* larvae was evaluated at ambient and elevated temperature for a subset of the sperm treatments above. Specifically, *P. daedalea* eggs from the Great Barrier Reef (one colony from Pelorus Island) were fertilized with local fresh, local cryopreserved, and Persian Gulf (Saadiyat Reef) cryopreserved sperm (Table 1). Embryos from each cross were reared to the planula stage of development in 3.5 l containers filled with 0.2 µm filtered seawater at ambient temperature (27 °C) with daily water exchanges. Larvae were pipetted into replicate scintillation vials (*n* = 10–20 larvae in 20 ml of 0.2 µm filtered seawater) and divided among ambient and elevated temperature treatments of 27 °C and 33  °C, respectively (*n* = 3 vials per sperm × temperature treatment). Temperature control was maintained in water baths (containers with titanium aquarium heaters and submersible pumps for circulation) and the elevated temperature treatment was ramped from 27 °C at a rate of 2 °C per hour. Water was changed in replicates immediately after survival counts were performed at 12, 36, 60, 84, and 108 h. During survival monitoring, larvae were counted as alive if they had an intact ectodermal wall, as larvae begin to lyse soon after death (Yakovleva et al., 2009). All embryos and larvae were destroyed following the last monitoring period.

A follow-up experiment was performed in May 2015 to assess whether cryoprotectant exposure affected larval survival. *P. daedalea* eggs from the Persian Gulf (three colonies from Saadiyat Reef) were fertilized with local fresh sperm without or with the addition of 20% DMSO for the fertilization period (*i.e.,* final concentration of 0.1% DMSO exposure for 4 h) and embryos were reared to the planula stage of development as above. Crosses were also set up with local cryopreserved sperm but ≤ 3 larvae per colony were produced due to low fertilization success. Individual larvae from fresh sperm treatments (*i.e.,* with and without DMSO) were pipetted into replicate wells of 96-well plates (*n* = 15–24 larvae each in 300 µl of filtered seawater) and divided among temperature treatments of 27 °C and 33 °C (*n* = 4 plates per sperm × temperature treatment). Temperature control was maintained in incubators and the elevated temperature treatment was ramped from 27 °C at 2 °C per hour. Survival counts were performed at 66 and 186 h as described above.

The rapid heat stress profiles used in both experiments has previously been shown to differentiate the thermal tolerance of *P. daedalea* populations and individual crosses (Howells et al., 2016a; Howells et al., 2021). The elevated temperature of 33 °C is 2.5 °C above the daily maximum mean of the Great Barrier Reef population (https://www.aims.gov.au/docs/research/climate-change/climate-monitoring/sst.html), and 3 °C below the maximum of the Persian Gulf population (Howells et al., 2016a).

### Data analysis

Variation in fertilization and survival was analysed using general linear models. For the fertilization experiment, separate models were run to evaluate the contributions of sperm treatment and source on the percentage of eggs fertilized. This was necessary because of the unbalanced experimental design where local populations were represented in both fresh and cryopreserved sperm treatments whereas foreign populations were only represented in the cryopreserved sperm treatment. Egg donor colony was included as a fixed factor, and both models were performed on log (*x* + 1) transformed data to improve normality and homogeneity of variance. Differences in fertilization among sperm sources were examined with Tukey post-hoc contrasts across all egg donors and within each colony. For the initial survival experiment, a single mixed-effect model was performed on *x*^2^ transformed data with sperm source and temperature as fixed factors and time as a random factor. For the follow-up survival experiment, a single model was performed with sperm source and temperature as fixed factors. For both survival experiments, differences among sperm sources/treatments within elevated temperatures were examined with Tukey post-hoc contrasts. These analyses were performed in R and the script and input data files are available Supplemental Information.

## Results

### Fertilization

**Figure 1:**
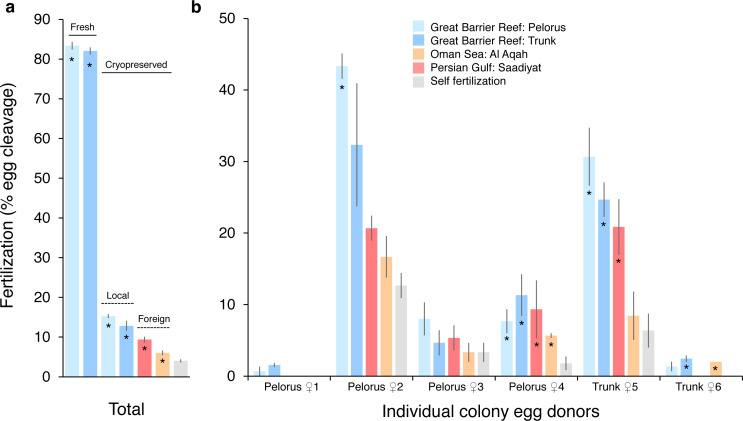
Fertilization of *Platygyra daedalea* eggs (mean % ± standard error) from the central Great Barrier Reef with fresh and cryopreserved sperm sources from local (Pelorus Island; Trunk Reef) and foreign (Al Aqah Island, Oman Sea; Saadiyat Reef, Persian Gulf) conspecific populations. (A) Overall rates of fertilization across egg donor colonies (*n* = 6), indicating significant contrasts between fresh and cryopreserved sperm (solid lines), local and foreign cryopreserved sperm (dashed lines), and outcrossing (asterisks) *versus* self-fertilization. (B) Variation in rates of fertilization among individual egg donor colonies from Pelorus Island (donors 1–4 from left to right) and Trunk Reef (donors 5–6), indicating significant contrasts between cryopreserved sperm sources *versus* self-fertilization (*). Contrasts were obtained from general linear model Tukey post-hoc comparisons in Tables S1 and S2.

The fertilization success of *P. daedalea* eggs from the Great Barrier Reef was strongly affected by sperm cryopreservation treatment (effect size [*η*^2^] = 0.89, *p* < 0.001; Table S1), to a lesser extent by sperm source (*η*^2^ = 0.54, *p* < 0.001; Table S2A), and further dependent on egg donor colony (*η*^2^ = 0.72–0.90, *p* < 0.001) (Fig. 1). In one egg donor colony, <5% of eggs were fertilized in all sperm treatments, and this data was excluded from statistical analysis. In local fresh sperm treatments (*i.e.,* Pelorus Island and Trunk Reef), 83% of eggs were fertilized on average, and ranged from 55% to 99% per egg donor colony. This is 5.9 and 10.8 times higher than average fertilization with local (13–15%) and foreign (6–9%) cryopreserved sperm sources, respectively, which also differed from one another (*p* ≤ 0.01; Table S2B) (Fig. 1A). In local cryopreserved sperm treatments, low to moderate fertilization was observed across egg donor colonies (Pelorus Island: 1–43%; Trunk Reef: 2–32%) (Fig. 1B). In foreign cryopreserved treatments, low fertilization was observed in 4–5 egg donor colonies (Persian Gulf: 0–21%; Oman Sea: 0–17%). Overall fertilization with all cryopreserved sperm sources was statistically greater than instances of self-fertilization (0–13% per egg donor colony) (*p* < 0.05; Table S2B), but these did not always differ at the level of individual egg donors (Table S2C). Self-fertilized embryos had a deformed appearance, did not develop into planula larvae, and died within 48-96 h. In contrast, a small number of larvae in monitored crosses had undergone settlement and metamorphosis at this time (*i.e.,* 4 of 5 larvae with fresh sperm; 0 of 5 larvae with local cryopreserved sperm; and 2 of 5 larvae with Persian Gulf cryopreserved sperm).

### Survival

**Figure 2:**
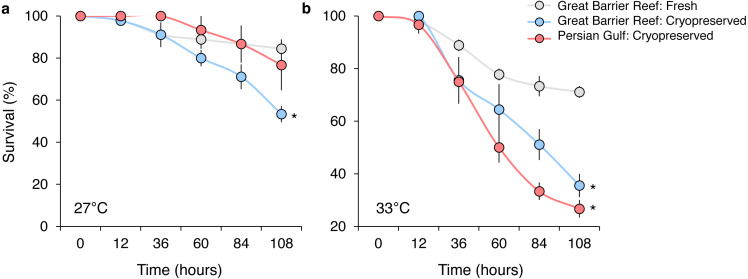
Survival of *Platygyra daedalea* larvae (mean % ± standard error) bred from central Great Barrier Reef eggs and either fresh local (Pelorus Island), fresh cryopreserved (Pelorus Island), or Persian Gulf (Saadiyat Reef) cryopreserved sperm at 27 °C (A) and 33 °C (B). Significant contrasts relative to local fresh sperm are indicated with asterisks (*) and were obtained from general linear mixed model Tukey post-hoc comparisons in Table S3.

In the initial experiment, the survival of *P. daedalea* larvae from the Great Barrier Reef was moderately affected by temperature (${\eta }_{p}^{2}=0.33$, *p* < 0.001), sperm source (${\eta }_{p}^{2}=0.12$, *p* < 0.001), and their interaction (${\eta }_{p}^{2}=0.12$, *p* < 0.001) (Table S3A). Predictably, overall end-point (108 h) survival at elevated temperature (44% at 33 °C) was 1.6 times lower than at ambient temperature (71% at 27 °C), but was further reduced with cryopreserved sperm sources (Fig. 2). At 27 °C, survival was 1.6 times lower for larvae bred with local cryopreserved sperm (53%) compared with larvae bred with fresh local sperm (84%) (*p* = 0.013; Table S3B), but was not reduced with foreign cryopreserved sperm (Fig. 2A). Survival impacts were exacerbated at 33 °C, where survival rates of larvae bred with local (36%) and foreign cryopreserved sperm (27%) did not differ from one another, but were 2.0 and 2.6 times lower than fresh sperm (71%), respectively (*p* < 0.005; Table S3B) (Fig. 2B).

**Figure 3:**
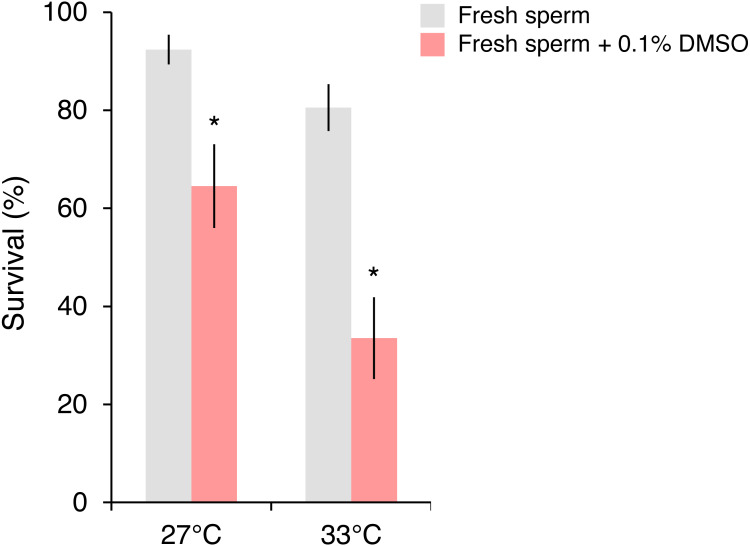
Survival of *Platygyra daedalea* larvae (mean % ± standard error) bred from Persian Gulf eggs and local fresh sperm either without or with cryoprotectant added (0.1% DMSO) during the fertilization period (4 h) at 27 °C and 33 °C. Significant contrasts between sperm treatments are indicated with asterisks (*) and were obtained from general linear mixed model Tukey post-hoc comparisons in Table S4.

In the follow-up experiment, the survival of *P. daedalea* larvae from the Persian Gulf was strongly affected by cryoprotectant exposure during fertilization (${\eta }_{p}^{2}=0.57$, *p* < 0.001), and moderately affected by temperature (${\eta }_{p}^{2}=0.19$, *p* = 0.007) (Table S4A). At 27 °C, survival of larvae exposed to 0.1% DMSO (65%) was 1.4 times lower than non-exposed larvae (92%; *p* = 0.012; Table S4B; Fig. 3). As with the initial experiment, the survival impact was greater at 33 °C, where the survival of exposed larvae (34%) was 2.4 times lower than non-exposed larvae (81%; *p* < 0.001).

## Discussion

This study provides the first demonstration of cross-breeding conspecific coral populations across ocean basins. Our results show that coral eggs from Great Barrier Reef populations of *P. daedalea* can be fertilized by cryopreserved sperm from distant populations from the Persian Gulf and Oman Sea. However, despite these sperm sources originating from environments up to 6  °C warmer than the Great Barrier Reef populations, we did not observe any gains in the thermal tolerance of cross-bred larval offspring. Sperm origin effects were most likely obscured by a strong negative effect of cryoprotectant exposure on larval survival. These findings highlight the importance of further optimization of cryopreservation and/or fertilization protocols to advance the application of sperm cryopreservation to selective breeding for trait enhancement.

### Thermal tolerance was not enhanced by cryopreserved sperm from warmer locations

*P. daedalea* in the southern Persian Gulf is adapted to sea temperatures that are not expected to occur on the Great Barrier Reef until the next century under the worst-case emissions scenarios (Howells et al., 2016a). Temperatures which remain above 34 °C for several consecutive weeks and reach highs of 36  °C in summer months have selected for genetic (and epigenetic) variants that confer heat tolerance in the *P. daedalea* population (Howells et al., 2021; Liew et al., 2020; Smith et al., 2022). These variants can be readily transferred to the cooler Oman Sea (Indian Ocean) population by cross-breeding with fresh sperm, where the resulting larval offspring had equivalent survival to Persian Gulf purebreds when exposed to heat (Howells et al., 2021). This is in contrast to the diminished survival under heat observed when the Great Barrier Reef population was cross-bred with cryopreserved sperm from the Persian Gulf, and most likely reflects cryoprotectant toxicity rather than outbreeding depression.

Outbreeding depression is a reduction in fitness that can occur in cross-breeding between genetically divergent populations. It is a risk of selective breeding that may not appear until subsequent generations (*i.e.,* F_2_ or later) (Edmands, 2007) and is generally expected to be low when selective breeding designs encompass populations with contemporary gene flow (Frankham et al., 2011). However, no contemporary gene flow is expected to occur between Persian Gulf and Great Barrier Reef populations of *P. daedalea* given the ∼12,000 km between sites and the high genetic divergence observed between Indian and Pacific Ocean populations of other coral species (Richards, Berry & Van Oppen, 2016). Marine populations in these ocean basins diverged from one another ∼1-3 million years ago during periods where lower sea levels restricted water flow between the Indian and Pacific Oceans (Benzie, 1999). Consequently, while outbreeding depression is a conceivable risk in our selective breeding design, it is not likely to explain the poor survival of cross-bred larvae as those sired by local cryopreserved sperm had comparably poor heat survival.

Cryoprotectants are essential to eliminate ice formation when cooling cells to cryogenic temperatures but have concentration, exposure, tissue, and species-specific toxic effects which present ongoing challenges to cryopreservation success (Best, 2015; Cirino et al., 2019). In corals, DMSO is the most widely used cryoprotectant and has generally been shown to have lower toxicity to coral sperm than alternatives (*e.g.*, polypropylene glycol, ethylene glycol, glycerol, methanol) but sensitivity varies among coral species (Hagedorn et al., 2012a; Hagedorn et al., 2006; Tsai et al., 2014; Viyakarn et al., 2018). Fertilization success in corals is also negatively impacted by DMSO exposure (Hagedorn et al., 2017; discussed in the following section), however, effects of cryoprotectants on later developmental states have not been explicitly tested. Our results of reduced larval survival in local (GBR) and distant (Persian Gulf) thawed sperm treatments (by 0–62%) and in an independent DMSO dosage experiment (by 36–71%) demonstrate that the toxic effects of DMSO continue beyond exposure during fertilization (0.1%, ∼2 h).

Evidence for longer-term impacts of DMSO exposure from other studies is mixed and suggestive of concentration- and/or species-specific effects. No sign of impacts to settlement and post-settlement survival were observed in *Acropora* corals bred with cryopreserved sperm under similar or lower concentrations of DMSO exposure compared with this study (*A. millepora* and *A. tenuis*, 0.02–0.08% DMSO, Hagedorn et al., 2017; *A. palmata*, 0.03–0.1% DMSO, Hagedorn et al., 2021). However, the settlement and post-settlement survival of *Diploria labyrinthiformis* bred with cryopreserved sperm under higher concentrations of DMSO exposure (0.2%for 2 h) was reduced by 17% and 8% relative to fresh sperm (Grosso-Becerra et al., 2021). Understanding the implications of DMSO toxicity for breeding programs using cryopreserved sperm requires longer-term monitoring of the survival and stress tolerance of coral offspring. For example, it is unclear whether the synergistic effect of elevated temperature and DMSO toxicity we observed in the larval stage of *P. daedalea* persists beyond settlement and establishment of symbiosis with photosynthetic dinoflagellates (Symbiodiniaceae).

### Fertilization success was impacted by sperm cryopreservation and origin

Variable and reduced fertilization observed with cryopreserved *versus* fresh sperm sources is commonplace in corals due to reductions in the motility of sperm by the cryopreservation procedure. In our study, *P. daedalea* sperm motility declined from 50–90% in samples observed immediately prior to cryopreservation to only 5% in thawed samples used for fertilization assays. This decline in motility is greater than has been reported elsewhere (Grosso-Becerra et al., 2021; Hagedorn et al., 2006; Hagedorn et al., 2017; Hagedorn et al., 2021; Ohki et al., 2014; Viyakarn et al., 2018) and reduced the effective sperm density to sub-optimal levels for fertilization in corals (*i.e.,* 5% of 10^5^–10^6^; see Nozawa, Isomura & Fukami, 2015). Accordingly, the fertilization values we observed in *P. daedalea* with local cryopreserved sperm sources (1–43% cryopreserved *vs.* 55–99% fresh) are in the lower range reported for other species: *Acropora digitifera* (>1–50% *vs.* >70–90%; Ohki et al., 2014), *A. humilis* (45% *vs.* 86%; Viyakarn et al., 2018), *A. millepora* (>50% *vs.* >90%; Hagedorn et al., 2017), *A. palmata* (37–82% *vs.* 91–99%; Hagedorn et al., 2021), *A. tenuis* (<25% *vs.* >90%; Hagedorn et al., 2017), and *Diploria labyrinthiformis* (0–90% *vs.* 30–90%; Grosso-Becerra et al., 2021).

Despite sperm motility being equally poor among thawed *P. daedalea* sperm sources, fertilization was lower with sires from distant (0–21%) *versus* local (1–43%) populations. This raises the possibility of a degree of gamete incompatibility between the Great Barrier Reef and the Middle East populations (Lobov et al., 2019). However, this remains unverified, especially as *P. daedalea* eggs from the Great Barrier Reef population are readily cross-fertilized by the sperm of several congeneric morphospecies (Miller & Babcock, 1997; Willis et al., 1997). Similarly, when Hagedorn et al. (2021) cross-bred isolated populations of *A. palmata* across a barrier to gene flow in the Caribbean, fertilization was low with distant (10–19%, Florida; 0–24%, Puerto Rico) relative to local (37–82%, Curaçao) cryopreserved sperm sources. However, the authors note that these values may not necessarily reflect incompatibility due to isolation, as fertilization success varies widely among *A. palmata* genets (Baums et al., 2013). An alternative explanation is that the quality of the Middle Eastern sperm sources was lower than those on the Great Barrier Reef (Hagedorn et al., 2016). While reproduction of the *P. daedalea* population in the Persian Gulf was impacted by a bleaching event in the same year that sperm was cryopreserved (Howells et al., 2016b), this bleaching event did not impact the Oman Sea population which had the lowest fertilization success. This explanation is also not supported by the pre-freeze sperm motility which was similar between Middle East and Great Barrier Reef sources.

The low occurrences of self-fertilization we observed in *P. daedalea* (0–13%) are consistent with those previously documented in *Platygyra* on the Great Barrier Reef (0–14%; Miller & Babcock, 1997). While self-fertilized zygotes did not survive beyond the early stages of embryogenesis, they potentially contributed to the values of fertilization recorded in cryopreserved sperm treatments. This emphasizes the importance of genetic testing to verify parentage in fertilization and selective breeding studies (see Hagedorn et al., 2021). While our biosecurity permit did not allow coral embryos to be preserved for downstream analyses, genetic testing should ideally be undertaken in studies where selfing occurs and/or when sperm-free controls are not utilized.

Finally, when breeding with cryopreserved sperm, it is desirable to maximize densities of thawed sperm to offset poor motility, but this results in a trade-off with cryoprotectant toxicity. Limited testing of the effects of DMSO exposure on fertilization has shown mixed results. In *Acropora millepora*, fertilization with thawed sperm was highest when the sperm-to-egg ratio was maximized (150,000:1) and the cryoprotectant concentration was minimized (0.02% DMSO) (Hagedorn et al., 2017). However, in the fertilization of *Lobactis* (formerly *Fungia*) *scutaria* with thawed sperm, the benefits of increasing egg-to-sperm ratio outweighed any negative effects of cryoprotectant exposure (up to 0.4% DMSO) (Zuchowicz et al., 2021a). In this study on *P. daedalea*, we used total sperm-to-egg ratios of ∼90,000:1 to ∼300,000:1 spanning the optimal range for *A. millepora*, but the effective ratios would have been greatly reduced by low post-thaw sperm motility (5%). The DMSO concentration of 0.1% was at a level which did not affect fertilization in *L. scutaria* (DMSO controls; Zuchowicz et al., 2021a), but was 5 times higher than the optimal concentration for fertilization in *A. millepora* (Hagedorn et al., 2017). Thus, cryoprotectant toxicity may also have contributed to the low fertilization success we observed in *P. daedalea*, yet further work is required to disentangle any effect from low effective sperm-to-egg ratios.

### Future directions

Since these experiments were conducted, the field of coral cryopreservation and reproduction has advanced significantly. For example, with new standardized methods for cryopreserving and freezing sperm (Zuchowicz et al., 2021b) and analysing sperm motility (Zuchowicz et al., 2021a). However, the application of cryopreservation for selective breeding requires further species-specific optimization of protocols to overcome bottlenecks to fertilization success, survival, and stress tolerance.

The use of DMSO as a cryoprotectant in this study and the concentration and exposure times used during fertilization were based on the best available knowledge from research in other coral species and there is scope for improvement. Subsequent testing of *P. daedalea* sperm with lowered DMSO concentration (5%) and alternative cryoprotectants (5–10% glycerol, 0.01–0.1 M mannitol) produced negligible improvements in the motility of thawed sperm compared with the 10% DMSO we used here (and resulted in poorer motility for *Acropora downingi*; Fig. S1). However, preliminary tests with supplementary sugars showed increases in post-thaw sperm motility of up to 40% for *P. daedalea* (12% on average) and 60% of *A. downingi* (50% on average). Sugar supplementation can protect cells from osmotic shock and maintain their functional integrity during cryopreservation and has previously produced promising results in *A. digitifera* (Ohki et al., 2015; Ohki et al., 2014). Post-thaw sperm motility could potentially be improved further by using ammonium chloride to activate motility (Ohki et al., 2014). To reduce cryoprotectant toxicity, sperm should be frozen at the highest possible density to allow for minimal concentrations to be used during fertilizations (Hagedorn et al., 2012a). Furthermore, exposure times could be reduced (especially with sperm activation) from the two hours used here as gamete contact times of 10-30 min are typically sufficient for high fertilization in corals (Nozawa, Isomura & Fukami, 2015). For example, in *L. scutaria*, fertilization success did not increase beyond 15 min of exposure to thawed sperm (M Hagedorn, 2022, unpublished data).

In addition to incorporating improvements in methodology, future assessments of the use of cryopreserved sperm for selective breeding may be more successful if they focus on cross-breeding at sub-regional scales. In this study, the large thermal gradient between the Persian Gulf and Great Barrier Reef coral populations provided opportunity to test for maximum gains in offspring heat tolerance. However, considerable standing genetic variation for thermal tolerance exists within regional metapopulations including the Great Barrier Reef (reviewed in Howells, Bay & Bay, in press) which can be harnessed for selective breeding (Dixon et al., 2015; Quigley et al., 2020). Selective breeding with cryopreserved sperm within metapopulations will allow signals of trait enhancement to be evaluated without potential negative outcomes from gamete incompatibility and/or outbreeding depression. Future studies should ideally also incorporate longer-term tracking of the performance of coral offspring (*i.e.,* at larval, post-settlement, and symbiotic stages) to provide a more comprehensive evaluation of the applications of cryopreservation technology to assisted evolution in corals. Yet, even with major technical advances, testing and implementation of assisted evolution and restoration initiatives remains resource intensive and feasible for only a targeted subset of coral populations, species, and locations. This underscores the importance for urgent action on climate change to limit the rate and magnitude of warming which will benefit all coral populations.

## Supplemental Information

10.7717/peerj.13395/supp-1 Supplemental Information 1 Supplementary Tables and Figures

10.7717/peerj.13395/supp-2 Supplemental Information 2 Data analysis

## Additional Information and Declarations

### Field Study Permissions

The following information was supplied relating to field study approvals (*i.e.*, approving body and any reference numbers): Permits for coral collection were provided by the Environment Agency Abu Dhabi, the Fujairah and Dibba municipalities, and the Great Barrier Marine Park Authority (G12/35236.1). Sperm samples were imported into Australia under CITES permits 13MEW5563 and PWS2013-AU-001007, and used for breeding in a quarantine approved research facility under Department of Agriculture, Fisheries and Forestry permit IP13016887.

### Data Availability

The following information was supplied regarding data availability: The raw data and R code are available in the Supplementary Files.
