# Supplementary material for: Challenges of sperm cryopreservation in transferring heat adaptation of corals across ocean basins
Source: PeerJ. 2022 May 27;10:e13395. doi: 10.7717/peerj.13395 (PMC9150692; doi:10.7717/peerj.13395)
Supplement: Supplemental Information 1 [file peerj-10-13395-s001.pdf]

Can heat adaptation be transferred across ocean basins?

Emily J. Howells, Mary Hagedorn, Madeleine J. H. van Oppen, John A. Burt

Supplementary Tables and Figures

Table S1. Analysis of variance table of the effects of sperm treatment (i.e., fresh versus cryopreserved) and egg donor colony on fertilization rates in *Platygyra daedalea*. Data were log (x +1) transformed to improve normality and homogeneity of variance.

Source	df	SS	MS	F	p	η_p^2	η^2
Sperm treatment	1	154.688	154.688	736.366	< 0.001	0.598	0.885
Egg donor colony	5	52.368	10.474	49.858	< 0.001	0.202	0.722
Sperm treatment × egg donor	5	31.424	6.285	29.918	< 0.001	0.121	0.609
Residuals	96	20.167	0.21				

Table S2a. Analysis of variance table of the effects of cryopreserved sperm source and egg donor colony on fertilization rates in *Platygyra daedalea*. Data were log (x +1) transformed to improve normality and homogeneity of variance.

Source	df	SS	MS	F	p	η_p^2	η^2
Sperm source	4	12.842	3.211	17.343	< 0.001	0.100	0.536
Egg donor colony	5	96.975	19.395	104.769	< 0.001	0.755	0.897
Sperm source × egg donor	20	7.492	0.375	2.023	0.019	0.058	0.403
Residuals	60	11.107	0.185				

Table S2b. Tukey post-hoc contrasts of overall cryopreserved sperm population effects on fertilization rates in *Platygyra daedalea*.

Contrast	t	p
Al Aqah - Nil	2.943	0.036
Al Aqah - Pelorus	-3.831	0.003
Al Aqah - Saadiyat	-0.821	0.923
Al Aqah - Trunk	-4.203	0.001
Nil - Pelorus	-6.774	< 0.001
Nil - Saadiyat	-3.764	0.003
Nil - Trunk	-7.146	< 0.001
Pelorus - Saadiyat	3.010	0.030
Pelorus - Trunk	-0.371	0.996
Saadiyat - Trunk	-3.381	0.011

Table S2c. Tukey post-hoc contrasts of cryopreserved sperm source effects on fertilization rates in *Platygyra daedalea* for each egg donor colony.

Egg donor colony	Contrast	*t*	*p*
Pelorus ♀1	Al Aqah - Nil	0.000	1.000
	Al Aqah - Pelorus	-1.042	0.835
	Al Aqah - Saadiyat	0.000	1.000
	Al Aqah - Trunk	-2.631	0.077
	Nil - Pelorus	-1.042	0.835
	Nil - Saadiyat	0.000	1.000
	Nil - Trunk	-2.631	0.077
	Pelorus - Saadiyat	1.042	0.835
	Pelorus - Trunk	-1.588	0.511
	Saadiyat - Trunk	-2.631	0.077
Pelorus ♀2	A lAqah - Nil	0.700	0.956
	Al Aqah - Pelorus	-2.692	0.067
	Al Aqah - Saadiyat	-0.640	0.968
	Al Aqah - Trunk	-1.649	0.473
	Nil - Pelorus	-3.391	0.010
	Nil - Saadiyat	-1.340	0.668
	Nil - Trunk	-2.348	0.144
	Pelorus - Saadiyat	2.052	0.255
	Pelorus - Trunk	1.043	0.834
	Saadiyat - Trunk	-1.009	0.850
Pelorus ♀3	Al Aqah - Nil	0.000	1.000
	Al Aqah - Pelorus	-2.114	0.228
	Al Aqah - Saadiyat	-1.042	0.835
	Al Aqah - Trunk	-0.723	0.950
	Nil - Pelorus	-2.114	0.228
	Nil - Saadiyat	-1.042	0.835
	Nil - Trunk	-0.723	0.950
	Pelorus - Saadiyat	1.072	0.820
	Pelorus - Trunk	1.391	0.636
	Saadiyat - Trunk	0.319	0.998
Pelorus ♀4	Al Aqah - Nil	2.959	0.034
	Al Aqah - Pelorus	-0.634	0.969
	Al Aqah - Saadiyat	-0.613	0.972
	Al Aqah - Trunk	-1.576	0.519
	Nil - Pelorus	-3.593	0.006
	Nil - Saadiyat	-3.572	0.006
	Nil - Trunk	-4.535	< 0.001
	Pelorus - Saadiyat	0.020	1.000
	Pelorus - Trunk	-0.942	0.879
	Saadiyat - Trunk	-0.962	0.871
Trunk ♀5	Al Aqah - Nil	0.423	0.993
	Al Aqah - Pelorus	-3.945	0.002
	Al Aqah - Saadiyat	-2.843	0.046
	Al Aqah - Trunk	-3.367	0.011
	Nil - Pelorus	-4.368	< 0.001
	Nil - Saadiyat	-3.266	0.015
	Nil - Trunk	-3.790	0.003
	Pelorus - Saadiyat	1.101	0.805
	Pelorus - Trunk	0.578	0.978
	Saadiyat - Trunk	-0.524	0.985
Trunk ♀6	Al Aqah - Nil	3.127	0.022
	Al Aqah - Pelorus	1.042	0.835
	Al Aqah - Saadiyat	3.127	0.022
	Al Aqah - Trunk	-0.349	0.997

	Nil - Pelorus	-2.085	0.240
	Nil - Saadiyat	0.000	1.000
	Nil - Trunk	-3.476	0.008
	Pelorus - Saadiyat	2.085	0.240
	Pelorus - Trunk	-1.391	0.636
	Saadiyat - Trunk	-3.476	0.008

Table S3a. Analysis of variance table of the effects of sperm source and temperature on survival rates in *Platygyra daedalea*. Time was included as a random factor and data were x^2 transformed to improve normality and homogeneity of variance.

Source	df	SS	MS	F	*p*	η_p^2	η^2
Sperm source	2,80	5.1E+07	2.5E+07	10.725	< 0.001	0.117	0.211
Temperature	1,80	1.4E+08	1.4E+08	60.443	< 0.001	0.33	0.43
Sperm source × temperature	2,80	5.1E+07	2.5E+07	10.656	< 0.001	0.116	0.21
Residuals	90	1.9E+08	2.1E+06				

Table S3b. Tukey post-hoc contrasts of sperm source effects on survival rates in *Platygyra daedalea* for each experimental temperature.

Temperature	Contrast	*t*	*p*
27°C	Pelorus cryopreserved – Pelorus fresh	-2.901	0.013
	Pelorus cryopreserved – Saadiyat cryopreserved	-3.648	0.001
	Pelorus fresh – Saadiyat cryopreserved	-0.746	0.737
33°C	Pelorus cryopreserved – Pelorus fresh	-3.491	0.002
	Pelorus cryopreserved – Saadiyat cryopreserved	1.689	0.216
	Pelorus fresh – Saadiyat cryopreserved	5.179	< 0.001

Table S4a. Analysis of variance table of the effects of DMSO sperm treatment and temperature on survival rates in *Platygyra daedalea*.

Source	df	SS	MS	F	*p*	η_p^2	η^2
DMSO	1	5.6E+03	5.6E+03	32.031	< 0.001	0.566	0.727
Temperature	1	1.8E+03	1.8E+03	10.473	0.007	0.185	0.466
DMSO × temperature	1	3.7E+02	3.7E+02	2.097	0.173	0.037	0.149
Residuals	12	2.1E+03	1.7E+02				

Table S4b. Tukey post-hoc contrasts of sperm DMSO treatment effects on survival rates in *Platygyra daedalea* for each experimental temperature.

Temperature	Contrast	*t*	*p*
27°C	No DMSO – DMSO added	2.978	0.012
33°C	No DMSO – DMSO added	5.026	< 0.001

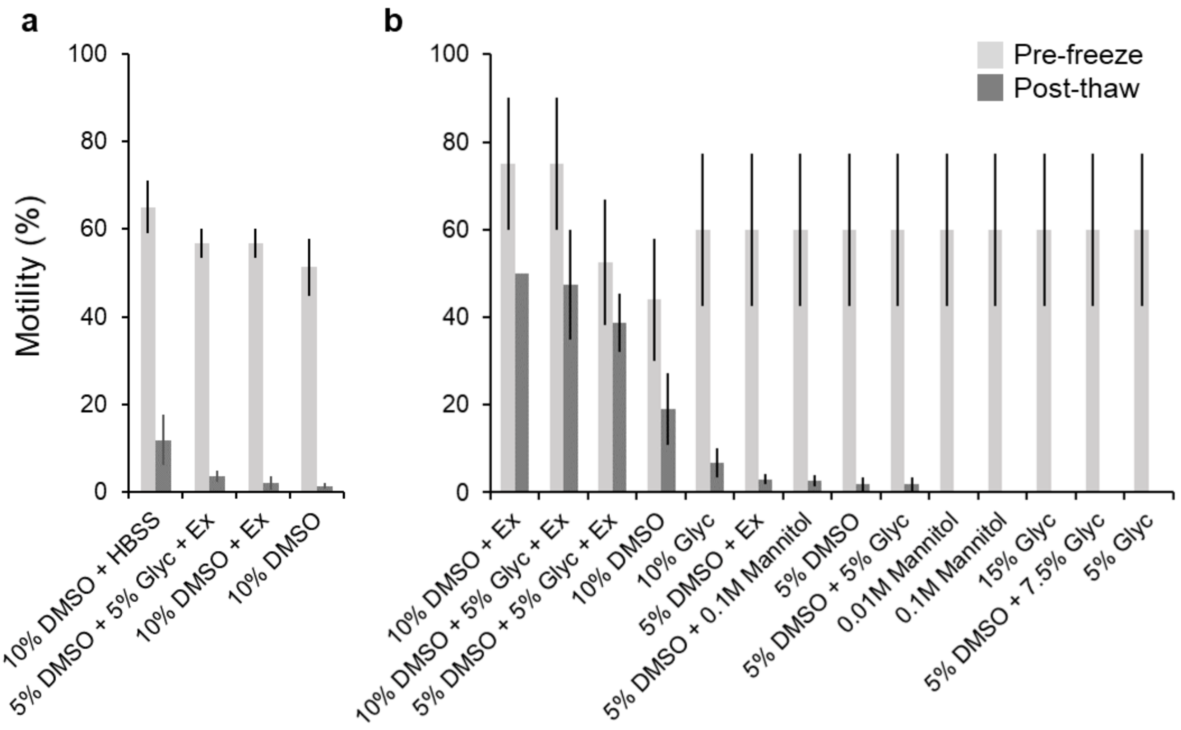

Figure S1. Pre-freeze and post-thaw motility of *Platygyra daedalea* (**a**) and *Acropora downingi* (**b**) sperm frozen with different cryoprotectants. Forward motility (mean % ± standard error) was evaluated in 1:10 dilutions of samples across spawning nights in between 2012 and 2014 (*n* = 3-8 and 2-5 spawning nights for *P. daedalea* and *A. downingi* per cryoprotectant treatment). Cryoprotectants used were dimethyl sulfoxide (DMSO), mannitol, glycerol (Glyc), commercial dual-sugar extender (Ex; Exodus 120PL; 50% final concentration), and Hanks' Balanced Salt Solution (HBSS; 2.8mM glucose final concentration).
